# Circumcision Not a Minor Operation

**Published:** 1896-05

**Authors:** Howard Crutcher

**Affiliations:** Chicago, Illinois


### CIRCUMCISION NOT A MINOR OPERATION.

HOWARD CRUTCHER, M. D., CHICAGO, ILLINOIS.

Circumcision may not be a major operation, but it is certainly not to be classed with the minor operations. In the first place an anesthetic, local or general, is required; vessels are to be ligated, and some careful stitching is necessary to secure a good result. The bleeding is sometimes very troublesome, and is never a matter of indifference to the careful surgeon.

Circumcision is really an amputation, and with this view in mind can be dealt with precisely as any other operation of the kind. My method is as follows:

Apply an elastic constrictor near the base of the organ; inject from ten to thirty drops of a four per cent. solution of Cocaine into the soft tissues of the prepuce; wait about three minutes, meantime working the tissues between the thumb and fingers; when the solution is thoroughly worked into the tissues, remove the constrictor, draw the prepuce well forward, which act locates the vessels, then ligate all the vessels by passing a needle around them and tying above, the suture line to be well behind the intended line of incision; next stitch the spaces between the vessels, being careful not to include too much tissue in the suture. When all sutures are tied, split the foreskin on the dorsum, and carry the incision around the organ until the necessary tissue is amputated. The method of amputating all the tissue by one sweep of the scissors is not satisfactory. If any vessel has escaped preliminary ligature it should be secured without delay. The frænum sometimes gives a troublesome hemorrhage, and any oozing at this point must receive the most careful attention. Once I had a dangerous and nearly fatal hemorrhage from this vessel. The bleeding was at last controlled as follows: The point was seized with strong forceps and drawn up; a needle was passed through the centre and the mass tied from both sides. It had been tied before, but transfixion was necessary to prevent the ligature from slipping.

The operation as described applies, of course, to adults and to those children in whom no adhesions are present, or, if present, are easily broken down. In adhesive cases it is often necessary to make a free incision before any progress can be made. In no operation can iron-clad rules be laid down.

Some may think the precautions against hemorrhage unnecessary, as the bleeding is generally trivial, but my experience is that some serious and perhaps fatal hemorrhages arise from amputation of the foreskin. The tissues are so soft that little aid can be expected from the natural hemostatic forces. One day recently I was called hastily to stop a hemorrhage in an infant of ten months, whose family physician had amputated the foreskin at one sweep and ignored all other features of the operation, not even putting in a stitch. It is a fact that the operation is often done in this manner. No one would think seriously of amputating a finger in this way, and yet of the two operations circumcision is frequently the bloodier.

As a matter of record I put the following in print: At a social gathering a gentleman apologized to me for his sleepiness by saying that his boy, aged about a year, had been so nervous and fretful that the entire household had been robbed of rest on account of the youngest member's pulmonary activity.

"Why don't you circumcise him?" I asked.

"Oh! he has been some time ago. Our family doctor don't believe in operation, but he gives a medicine that has the same effect."

The name of this medicine, and especially the name of the doctor that gives it, ought to be made known. It is a pity that such astonishing wisdom should have remained concealed so long.

The removal of a gangrenous leg will not cure a patient, but it is very certain that a cure never can be brought about without amputation. Circumcision may not completely cure every case, but it certainly is an obstacle of great magnitude in many instances.—*Journal of Orificial Surgery*, February, 1896.

